# A Longitudinal Exploratory Study of SARS-CoV-2 Antibody Dynamics in Young Adults in Bogotá: Lessons from Natural Infection and Post-Vaccination Memory

**DOI:** 10.3390/biomedicines14040849

**Published:** 2026-04-08

**Authors:** María F. Naranjo-Ortíz, Luz Parada-Rubio, José Fuentes-Montoya, Jean Carlos Villamil Poveda, Francy Elaine Torres-Suarez, Heidy-C. Martínez-Díaz, Laura Daniela Ardila Ortiz, Juliana Velosa-Porras, Lorenza Jaramillo, Jorge Andrés Castillo, Jairo Jaime, Nelly S. Roa, Adriana P. Corredor-Figueroa

**Affiliations:** 1 Centro de Investigación en Inmunología e Infectología Veterinaria (CI3V), Facultad de Medicina Veterinaria y de Zootecnia, Departamento de Salud Animal, Universidad Nacional de Colombia, Sede Bogotá, Bogotá 111321, Colombia; mfnaranjoo@unal.edu.co (M.F.N.-O.); jjaimec@unal.edu.co (J.J.); 2 Laboratorio de Investigación en Ciencias Biológicas (LiCiB), Centro de Investigación e Innovación en Ciencia y Tecnología (CEINTECCI), Universidad ECCI, Bogotá 111311, Colombia; angeliquithaparada@gmail.com; 3 Grupo INDEVOS, Data Science Program, Faculty of Engineering, Universidad Ean, Bogotá 111311, Colombia; jamontoya.d@universidadean.edu.co; 4 Centro de Investigaciones Odontológicas, Faculty of Dentistry, Pontificia Universidad Javeriana, Bogotá 110231, Colombia; jean.villamil@javeriana.edu.co (J.C.V.P.); h-martinez@javeriana.edu.co (H.-C.M.-D.); juliana.velosa@javeriana.edu.co (J.V.-P.); lorenzaj@javeriana.edu.co (L.J.); 5 Centro de Investigación en Salud para el Trópico—CIST, Faculty of Medicine, Universidad Cooperativa de Colombia-Sede Santa Marta, Santa Marta 470004, Colombia; francy.torress@campusucc.edu.co; 6 Grupo de Investigación Armonía, Centro de Investigación y Atención Psicosocial Hanami, Bogotá 111221, Colombia; 7 Grupo de Enfermedades Infecciosas, Departamento de Microbiología, Facultad de Ciencias, Pontificia Universidad Javeriana, Bogotá 110231, Colombia; jandrescastillo@javeriana.edu.co

**Keywords:** COVID-19, SARS-CoV-2, neutralizing antibodies, seroprevalence, Bogotá-Colombia

## Abstract

**Background**: Infections caused by severe acute respiratory syndrome coronavirus 2 (SARS-CoV-2) have generated major public health concerns worldwide. Young adults represent a critical group for viral transmission due to their high proportion of asymptomatic infections. **Objective**: To characterize the dynamics of SARS-CoV-2-specific antibodies in individuals aged 20–29 years from Bogotá, Colombia, across two longitudinal phases. **Methods**: Phase I assessed seroprevalence, seroconversion, spatial clustering, symptoms associated with seropositivity and antibody kinetics following natural infection. Phase II evaluated vaccine-induced antibodies, immune memory, and neutralizing capacity. Analyses included Functional Principal Component Analysis, survival analysis, clustering, and predictive modeling. **Results**: In Phase I, a seroprevalence of 15.59% (17/109 participants enrolled) was observed, while seroconversion among those who completed all six sampling points was 30.18% (16/53), with clusters of positive cases in different areas of Bogotá. The symptoms most associated with seropositivity included mucus hypersecretion, fever, and respiratory difficulty. Antibody responses were heterogeneous: naturally infected individuals generally showed high titers during the first 1–2 months, remaining detectable up to 4 months. The reduction in dimensionality suggested dominant humoral patterns, and clustering revealed two immune profiles differing in the risk of seroconversion. Predictive modeling indicated diverse antibody trajectories over 12 months. In Phase II (2024), three long-term immune memory clusters (low, medium, high) were observed; post-vaccination IgG titers were observed, although in most cases they lacked neutralizing activity. **Conclusions**: This longitudinal exploratory observational study provides an initial characterization of antibody dynamics in young adults, suggesting their potential epidemiological relevance and offering preliminary insights into post-infection and post-vaccination immunity.

## 1. Introduction

The coronavirus responsible for severe acute respiratory syndrome (SARS-CoV-2) is a virus belonging to the *Coronaviridae* family (genus *Betacoronavirus*), whose members are enveloped viruses with a single-stranded RNA (ssRNA) genome [1].

SARS-CoV-2 was first identified in Wuhan, China, in 2019 and rapidly spread worldwide, causing one of the most severe pandemics in human history, COVID-19 [2]. By the end of 2023, approximately 670 million COVID-19 infections had been reported globally, resulting in more than 6.8 million deaths due to the infection [3,4]. In addition to its substantial impact on human health, the COVID-19 pandemic has affected healthcare systems, the global economy, and the mental health of individuals worldwide [5].

Transmission of SARS-CoV-2 occurs primarily through aerosols, either by direct contact with infected individuals or through contact with contaminated surfaces [6]. Several known risk markers are associated with COVID-19 morbidity and mortality, including age, gender, race and disease, such as hypertension, type 2 diabetes associated with obesity such as metabolic syndrome, and chronic cardiovascular diseases, blood groups A and AB, and a lower risk associated with living in remote, less crowded regions [7,8].

Since the appearance of SARS-CoV-2 in 2019, the virus has spread worldwide, reaching 497,960,492 confirmed cases and 6,181,850 deaths globally as of 12 April 2022, of which 6,088,034 confirmed cases and 139,719 deaths were reported in Colombia [9]. Particularly in Colombia, individuals between 20 and 29 years of age represent the second largest age group with confirmed infections, following those aged 30 to 39, with 1,278,670 nationwide and 384,585 cases in Bogotá (capital of Colombia) [10]. Accordingly, it has been reported that young people tend to exhibit asymptomatic SARS-CoV-2 infections and lower mortality rates due to viral infection. However, this age group may play a significant role in the transmission and spread of the virus since asymptomatic infection may contribute to an increase in transmission of the virus to hosts susceptible to the virus [11,12,13,14]. Furthermore, the contact rate between individuals can vary according to social characteristics and interactions, and these tend to increase among young individuals [15].

While antibody kinetics, waning immunity, and hybrid immunity to SARS-CoV-2 have been described in diverse populations worldwide, most longitudinal evidence has been generated in high-income settings and in cohorts with broad age ranges or clinical heterogeneity [16] limiting specific insights into age- and context-specific dynamics (e.g., antibody decay patterns, stabilization phases, and hybrid responses) reported in longitudinal cohorts elsewhere [17]. However, young adults from urban Latin American settings remain underrepresented in such longitudinal immunological studies, despite evidence that underlying social, economic and epidemiologic contexts can shape exposure, transmission and immune response [18]. Furthermore, few longitudinal studies have examined antibody dynamics in populations exposed during periods dominated by locally emerging variants with immune-evasive potential. This gap in evidence underscores the need for representative cohort studies to characterize age, specific antibody kinetics, waning patterns and hybrid immunity among young adults in Latin American urban environments [18].

Despite the success of vaccination campaigns during COVID-19 pandemic, the high mutation rates of SARS-CoV-2, particularly in the spike (S) gene, combined with its rapid transmission, have driven the emergence and spread of multiple variants, including B.1.351 (Beta), B.1.617.2 (Delta), B.1.1.529 (Omicron), B.1.621 (Mu) [19], B.1.1.7 (Alpha), and B.1.1.28-derived P.1 (Gamma) [20], among others. Increasing evidence suggests that these variants have significantly influenced viral pathogenicity, immunogenicity, and transmission dynamics, thereby enhancing viral spread and contributing to surges in COVID-19 case prevalence across different regions. In particular, the emergence of new variants has been associated with higher transmissibility and shifts in epidemiological patterns, underscoring their critical role in shaping the course of the pandemic [20]. For instance, the detection of the B.1.1.7 (Alpha) variant in Lebanon in late 2020 coincided with marked increase in COVID-19 cases, highlighting the impact of emerging variants on transmission dynamics and their broader public health implications [20].

In this context, seroprevalence studies, particularly those based on samples collected from the pandemic period, remain essential for providing insights into circulation of variants and their implications for the development and persistence of protective immune response. On the other hand, the Mu variant was shown to exhibit spike mutations associated with immune evasion during the pandemic, which raised concerns about its potential impact on vaccine efficacy and natural immunity [21]. Understanding antibody dynamics in the context of this specific variant is essential for accurately interpreting seroprevalence patterns and characterizing the development and persistence of humoral immune memory responses in young adult populations.

To date, seroconversion studies following natural infection and vaccination in young adult populations remain limited. Emerging evidence has shown that some age groups may play a significant role in shaping the antibody production dynamics of a population; for example, a cohort study in the United States found that individuals aged 19 to 30 years exhibited the lowest IgG levels among all age groups [16]. Similarly, longitudinal analysis of a cohort of a 22-year-old cohort showed a considerable decline in anti-spike IgG antibodies over a 7-month period following natural infection [22], highlighting the potential for waning humoral immunity in this population. Although seroprevalence estimates in Colombia was similar between children and young adults [23], the temporal dynamics of seroconversion following natural infection and vaccination in young adults during the pandemic remain insufficiently characterized. Furthermore, the epidemiological context of Colombia during the study period was marked by the circulation of multiple SARS-CoV-2 variants, including the Mu variant (B.1.621), which was first identified in Colombia in January 2021 and became the dominant strain during the first half of 2021 [24].

Several studies have described the dynamics in different populations (such as students, workers, or young adults); for example, in a cohort of university students (18–29 years) conducted in the United States (University of Wisconsin-Madison), seroconversion was observed over the course of a semester, followed by a more pronounced decline in nucleocapsid antibodies compared to anti-spike antibodies [25]. Other studies have shown that the magnitude and duration of the humoral response may vary according to age. For example, in a longitudinal study performed over 7 months among healthcare workers in Barcelona, Spain, lower and more fluctuating levels of anti-SARS-CoV-2 IgG were observed in young adults compared with elderly individuals [26]. Additionally, in a prospective longitudinal study undertaken in Casablanca, Morocco, with follow-up extending up to 12 months, different kinetic profiles of IgM, IgA, and IgG were described, with some young individuals showing a more rapid decline in antibody levels [27]. Other studies have also identified an association between age and antibody levels. For example, a prospective multicenter study implemented in hospitalized patients in the United Kingdom, reported that young adults may present lower sustained titers compared to other age groups [28]. Consistent with this evidence, a study carried out among healthy young adults in North Carolina, United States, reported a rapid peak in nucleocapsid IgG levels followed by a more pronounced decline in the following months [22]. Overall, these results highlight the importance to study antibody production dynamics in young adults within the Colombian context.

Although cross-sectional seroprevalence studies (IgG + IgM) have been conducted in several Colombian cities [23,29] among the general population, healthcare workers and students in Bogotá [30,31], as well as in SARS-CoV-2 vaccinated individuals [32] the available evidence on antibody kinetics in young adults (20–29 years) is limited.

In particular, there is a scarcity of longitudinal studies that may allow an understanding of how antibody levels against SARS-CoV-2 can vary in this age group, especially in scenarios involving re-exposure or reinfection. This lack of information restricts the ability to precisely characterize the immune response and its relationship with the epidemiology of infection in a population that, due to its high mobility and social activity, may play a relevant role in virus transmission.

Based on this framework and considering the limited information available on SARS-CoV-2 epidemiology in young adults in Colombia, the objective of this study was to describe the epidemiological behavior of SARS-CoV-2 in this population. To achieve this, a longitudinal exploratory study was conducted in two phases. In the first phase, over a six-month period (December 2020 to May 2021), antibody production against SARS-CoV-2 through natural exposure was evaluated in a population of individuals aged 20 to 29 years from the city of Bogotá, Colombia, identifying risk factors, signs and symptoms, seroprevalence, antibody dynamics, and incidence due to natural infection. In the second phase, antibody response was analyzed 36 months after the last sample collection of the first phase and following vaccination, which took place between 2021 and 2022, in a subset of participants from the first phase, with the aim of evaluating the vaccine induced memory response in this cohort and understanding its relationship with the natural history of the disease.

## 2. Materials and Methods

### 2.1. Study Design

**Figure 1:**
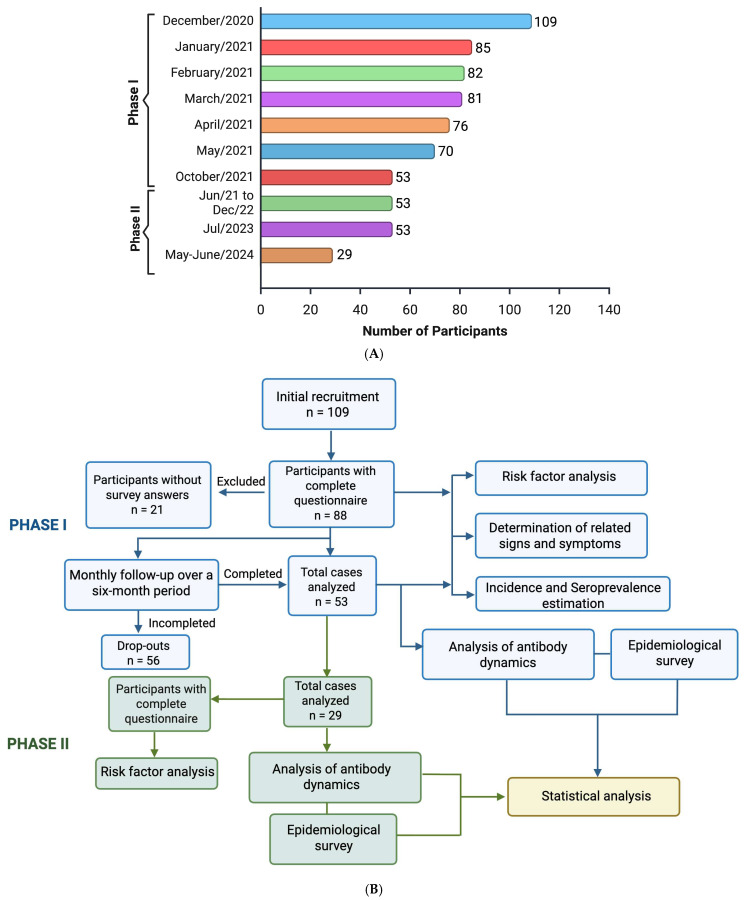
Schematic representation of the longitudinal exploratory study (Phases I and II) designed to evaluate the seroprevalence of antibodies against COVID-19 in individuals aged 20–29 years. (**A**) Phase I was conducted from December 2020 to October 2021, including data analysis. The timeline of the 6-month follow-up period shows the number of participants at each sampling point, with different colors indicating each monthly collection. Fifty-six participants had discontinuous follow-up during this period. Phase II timeline national vaccination program (June 2021–December 2022), active participant follow-up (June 2023–July 2024), and ELISA assays with subsequent data analysis in 2025. (**B**) This flowchart illustrates the study design according to the stated objectives. “Drop-out” was defined as failure to participate in one or more sampling points during the follow-up period. Created in BioRender.com. Roa NS. (2026) https://app.biorender.com/illustrations/canvas-beta/69235b9a62509eede4d26ec6 (accessed on 23 November 2025).

For this research, a longitudinal exploratory observational study with an analytical and correlational component was proposed. The study population was considered to be 1,460,836 individuals between 20 and 29 years of age from the city of Bogotá (Colombia) [33]. To achieve an adequate sample size for the planned analyses, the minimum sample size to ensure representativeness with respect to the target population was 78 individuals, which was determined using Winepiscope 2.0 software with a 99% confidence interval, 5% standard error, and an expected prevalence of 3% [14,34,35]. To increase precision and mitigate possible attrition during the follow-up process, a total of 109 participants were enrolled. Recruitment was conducted through a non-probabilistic convenience sampling strategy, primarily via institutional networks at the participating universities (Universidad ECCI, Pontificia Universidad Javeriana, and Universidad Nacional de Colombia) and social media outreach. It should be noted that this sampling approach does not permit probabilistic inference to the broader Bogotá population of this age group, and findings should be interpreted within the exploratory scope of the study, with age and city of residence (Bogotá) serving as inclusion criteria. Medical restrictions regarding peripheral blood sample collection (peripheral vascular disease, high risk of bleeding, or peripheral arterial disease) and residing in another city were considered as exclusion criteria. The study was designed in 2 phases (Figure 1A,B). A total of 53 cases were analyzed in the phase I, as these participants completed follow-up for all six sampling time points and completed the questionnaire. Phase II consisted of a cross-sectional follow-up study conducted two years later, in which the 53 original completers were invited to participate; 29 responded and were included in the study.

### 2.2. Sample Collection and Processing

Peripheral blood samples were collected by a trained healthcare professional using Vacutainer^®^ tubes (Franklin Lakes, NJ, USA) without anticoagulant and containing a separating gel. All samples were obtained under aseptic conditions, ensuring proper subject identification and appropriate handling of biological material. Following collection, the samples were maintained at controlled room temperature until complete coagulation was achieved (approximately 30 min). Once the clot had formed, serum was recovered by centrifugation at 1800 revolutions per minute for 10 min. The resulting serum was carefully transferred into clean Eppendorf tubes and stored at −20 °C until further processing. Sample collection was performed monthly for each participant over a six-month period, from December 2020 to May 2021, corresponding to Phase I. For Phase II, active participant follow-up and sample collection were conducted between June 2023 to July 2024, following the national vaccination program (June 2021–December 2022) and 36 months after the final sampling point of Phase I. ELISA assays and subsequent data analysis were performed in 2025 (Figure 1A).

Specific antibodies against SARS-CoV-2 were detected using an ELISA assay with the INgezim^®^ COVID-19 DR kit (Madrid, Spain; sensitivity 98.4% and specificity 99%), following the manufacturer’s instructions. This assay enables the detection of IgG, IgM, and IgA antibodies specific against the nucleocapsid (N) protein of SARS-CoV-2. Absorbance values were measured at 450 nm using a calibrated Biotek ELX800 microplate reader (Winooski, VT, USA). Optical density (OD) data from each sample was recorded in Microsoft Excel files for subsequent analysis. The cut-off value for the INgezim^®^ COVID-19 DR ELISA was calculated according to the manufacturer’s instructions using the formula:

Cut-off=Mean OD of Negative Controls+0.150

This calculation is performed for each plate, ensuring that the interpretation reflects the specific conditions of the assay. In our study, the cut-off obtained was approximately 0.710 OD, which was used to classify results as follows: samples with OD values greater than 0.710 were considered positive, while those below this threshold were classified as negative. According to the kit insert, a doubtful zone is defined for values close to the cut-off: Negative: OD < 0.9 × Cut-off; Doubtful: OD between 0.9 × Cut-off and 1.1 × Cut-off; Positive: OD > 1.1 × Cut-off. This approach confirms that the cut-off is not a fixed universal value but depends on the optical density of the negative controls measured in each run. Non-normalized OD data were used for the analysis, as all longitudinal samples from the same individual were processed under consistent assay conditions to preserve within-subject temporal trends. While inter-plate variability is a recognized limitation, all longitudinal samples from each participant were processed under consistent assay conditions, minimizing systematic measurement bias across time points.

For Phase II, the following kits were used: the Human SARS-CoV-2 Spike (trimer) Total Ig ELISA kit, catalog number BMS2323 (Invitrogen, Carlsbad, CA, USA); the Human SARS-CoV-2 Spike (trimer) IgM ELISA kit, catalog number BMS2324 (Invitrogen); and the SARS-CoV-2 Neutralizing Antibody ELISA kit, catalog number BMS2326 (Invitrogen). Samples were processed according to the manufacturer’s instructions. The OD (for qualitative values) was calculated using the ratio obtained from the absorbance of the calibrator divided by the absorbance of the sample. If the ratio was <1, the target immunoglobulin was considered absent; if the ratio was >1.3, the target immunoglobulin was considered present. Ratios between 1 and 1.3 were classified as indeterminate.

Total Ig antibody (IgG) titers were quantified using an indirect ELISA. When the High Control was included to generate a standard curve, optical density (OD) values were fitted using a four-parameter logistic (4-PL) regression model, which provided the optimal curve fit. Prior to curve fitting, background absorbance was subtracted from all measurements, including standards, controls, and unknown samples. Sample concentrations were interpolated from the standard curve and subsequently corrected by the corresponding dilution factor. Samples yielding OD values above the upper limit of quantification were further diluted in Standard Diluent Buffer and reanalyzed; final concentrations were multiplied by the additional dilution factor. For quantitative analysis, serum samples were pre-diluted 1:10 and subsequently diluted on the plate to reach a final dilution of 1:75,000.

ELISA measurements from Phase I (Anti-N antibodies) and Phase II (anti-Spike antibodies) were obtained and analyzed independently, as they target distinct antigens and were commercially available at different times across the study phases, precluding direct predictive comparisons between phases.

### 2.3. Survey and Data Collection

To identify potential risk factors and signs and/or symptoms associated with SARS-CoV-2 infection in the individuals evaluated, a survey was developed using the Google Forms platform to collect identification data and demographic, social, and health-related characteristics of each participant and their environment. The survey was administered at the time of enrollment of each study participant, yielding a total of 88 respondents. This procedure was applied in both Phase I and Phase II.

### 2.4. Definition of Variables

For the analysis of associated risk factors and signs and/or symptoms, the ELISA test result was used as a dichotomous dependent variable (positive or negative). For the analysis of antibody dynamics, the OD value was considered in both phases I and II according to the established cutoff; values above the threshold were classified as positive, whereas those below were considered negative. In phase II, quantitative measurements were also included, namely antibody concentrations expressed in Kunits/mL. Additionally, based on the information collected through the survey, independent variables were defined and classified into three groups: personal information, activities, and signs/symptoms and protective measures.

### 2.5. Statistical Analysis

For the statistical analysis, different subsets of the total sample were used according to each of the study objectives.

Attrition Bias Analysis: To evaluate the potential impact of participant dropout on study validity, a sensitivity analysis was conducted comparing baseline characteristics between participants who completed all six sampling points (completers, *n* = 53) and those with discontinuous follow-up (dropouts, *n* = 56). Demographic variables (sex, age, occupation) and risk factors were compared using chi-squared tests for categorical variables and Welch’s *t*-test for continuous variables.

Seroprevalence was determined as the percentage of positive cases based on the total number of recruited individuals (*n* = 109), using a descriptive statistical analysis. Subsequently, only participants who completed the survey were considered (*n* = 88) to identify potential risk factors and signs and/or symptoms associated with infection. Additionally, the seroconversion probability was estimated among participants included in the complete analysis of Phase I (*n* = 53).

The association between each independent variable and the different dependent variables was evaluated using a Logistic Regression Model and a Probit Regression Model with bivariate analysis, as well as parametric statistics using Pearson’s correlation coefficient (or Spearman’s rank correlation for non-parametric analysis). For variables with an Odds Ratio (OR) < 1 and a non-significant chi-squared value, Fisher’s Exact Test was applied to confirm the absence of a statistical relationship with SARS-CoV-2 infection. IBM SPSS^®^ Statistics (v25; IBM Corp., Armonk, NY, USA) and RStudio^®^ (v1.4.x; RStudio Inc./Posit PBC, Boston, MA, USA) were used for these analyses.

To describe the antibody dynamics during the follow-up period, only individuals who participated in all sampling sessions were included; those who missed one or more of the six sampling points were excluded.

All data processing and statistical analyses were performed using Python (v3.11.0) and R (v4.3.1). In Python, data manipulation was performed using pandas (v2.0.3), numerical computations were carried out with NumPy (v1.24.3), and statistical analyses were implemented using SciPy (v1.10.1). Machine learning tasks—including clustering, dimensionality reduction, and predictive modeling—were conducted using scikit-learn (v1.3.0). Functional Principal Component Analysis (fPCA) was implemented using the scikit-fda package, while survival analysis was performed using the lifelines library, based on the Kaplan–Meier estimator and the log-rank test. Data visualization was carried out using Matplotlib (v3.7.2) and seaborn (v0.12.2). In R, complementary analyses related to functional data analysis and survival modeling were performed using the fda and survival packages. The analysis was structured according to the two phases of the study.

### 2.6. Longitudinal Data Analysis (Phase I)

For Phase I, which included six monthly antibody measurements for 53 individuals, functional data analysis (FDA) techniques were employed to model the temporal dynamics of the immune response.

Functional Principal Component Analysis (fPCA): The antibody trajectories of each individual were treated as functional data. The goal of fPCA was to reduce the high dimensionality of the longitudinal dataset and identify the dominant modes of variation in the immune response curves. Discrete curves were smoothed using B-spline basis functions to generate continuous representations, after which fPCA was applied to these functions. The first two principal components (PC1 and PC2), which explained 67.2% and 17.0% of the total variance, respectively, were retained for subsequent analyses. These components represented the overall magnitude of the response (PC1) and its temporal change (PC2).

Clustering Analysis: To identify subgroups of individuals exhibiting similar immune response profiles, the K-Means clustering algorithm was applied to the scores of the first two principal components (PC1 and PC2). The optimal number of clusters (*k* = 2) was selected based on the maximization of the Silhouette Score, which reached its highest value (0.771) under this configuration, indicating the most robust and naturally separated partition of the data.

Predictive Analysis of Antibody Dynamics: To extend the visualization and interpretation of immunological dynamics beyond the six-month observation period, an exploratory predictive analysis was implemented using machine learning techniques for time series. The original matrix containing measurements for the first six months was transformed into a feature set, where the first four observations per individual were used to predict the fifth, and then months two to five predicted the sixth. This procedure was applied recursively to generate projected values up to month twelve. Predictor variables were standardized to ensure numerical homogeneity and prevent bias during model training. The selected model for prediction was 500 trees in a Random Forest with and a depth of six levels, chosen to capture non-linear patterns and remain robust against biological noise inherent to the data. Individual predictions were subsequently compared with observed curve segments (months 1–6) and projected segments (months 7–12) to evaluate model performance metrics. Due to the limited sample size and the recursive nature of the forecasting approach, this analysis was conceived as an exploratory framework to assist in the identification of general trajectory patterns rather than to provide precise individual-level predictions.

### 2.7. Cross-Sectional Data Analysis (Phase II)

For Phase II, which consisted of a single measurement per individual (*n* = 29) in 2024, the analytical approach was adapted to the cross- sectional nature of the data.

Clustering Analysis: The K-Means algorithm was applied directly to the standardized values of the main variable, Total Ig OD. As in Phase I, the optimal number of clusters (*k* = 3) was determined by maximizing the Silhouette Score (0.724), identifying three long-term response groups: Low, Medium, and High.

Association Analysis and Risk Factors (Both Phases, Analyzed Independently)

Association with Categorical Variables: The association with all 42 categorical variables that met data quality criteria was evaluated. The choice of statistical test depended on the number of categories within each variable:

Independent Student’s *t*-test: Applied to all dichotomous variables (two categories, e.g., Smoking: Yes/No) to compare the mean immune response biomarker between groups.

One-way Analysis of Variance (ANOVA): Applied to all polytomous variables (more than two categories, e.g., Occupation) to compare the mean biomarker levels across multiple groups.

This approach is justified as it represents the standard and most direct statistical method to compare the mean of a continuous variable across two or more independent groups. In all cases, a *p*-value < 0.05 was considered statistically significant.

Association with Continuous Variables: To assess the linear relationship between the immune response biomarker and other continuous variables (e.g., age, behavior scores), Pearson’s correlation coefficient (*r*) was calculated. The *p*-value associated with each correlation was used to determine the statistical significance of the association.

### 2.8. Ethical Considerations

This project was approved by the Research and Ethics Committee of Universidad ECCI and by the Ethics Committee of the School of Dentistry at Pontificia Universidad Javeriana (CIEFOPUJ), in accordance with institutional ethical oversight requirements at the participating institutions, including Universidad ECCI, Pontificia Universidad Javeriana, and Universidad Nacional de Colombia, where participant recruitment and sample collection were conducted under the supervision of the respective principal investigators. All participants completed an informed consent form at the time of enrollment in the study, in which the procedures for sample collection, storage, and processing were explained in detail, as well as the handling of the information gathered through the survey and during the follow-up period.

## 3. Results

### 3.1. Phase I

#### 3.1.1. Seroprevalence and Seroconversion Rates

**Figure 2:**
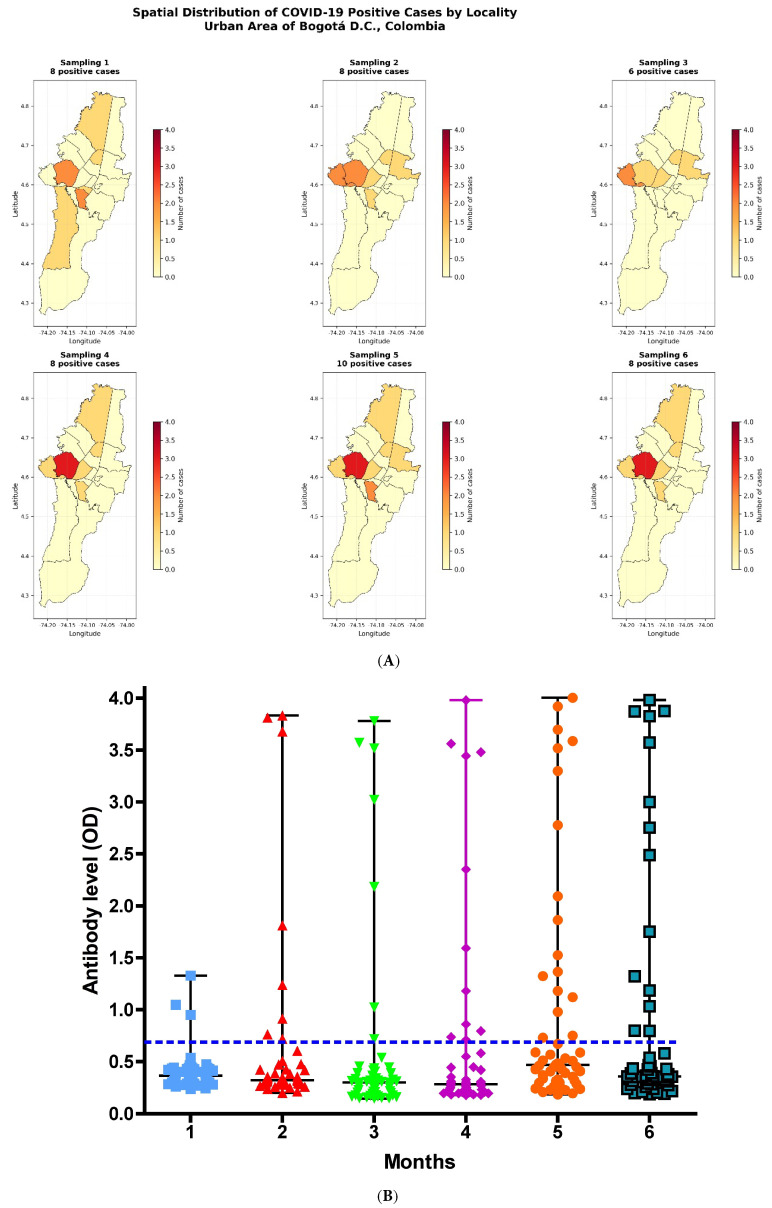
Spatial and temporal distribution by locality of individuals aged 20 to 29 years in Bogotá, Colombia, based on the presence of SARS-CoV-2-specific antibodies during a six-month follow-up period. (**A**) Positive cases by locality across each of the six-sampling rounds. Color intensity indicates the number or individuals with detectable SARS-CoV-2-specific antibodies in each locality at each sampling point, allowing a visualization of changes in geographic concentration over time. (**B**) Individual data for total anti-N antibody (OD) over the 6-month follow-up period. Each point represents the OD value for an individual, and the colors correspond to the sampling time points for each month, as described in Figure 1A. Data are presented as median and interquartile range (IQR) for each month. The dotted line indicates the cutoff value (OD > 0.710).

The seroprevalence was 15.59% (*n* = 109). Among these 109 individuals, regarding the geographical distribution within the city of Bogotá, most positive cases were reported in the southern area (localities of Ciudad Bolívar, Rafael Uribe, and Soacha) and the northwestern area (locality of Engativá) (Figure 2A). In the first sampling, a large number of positive clusters were observed across most peripheral localities of Bogotá. In the second, third, and fourth sampling rounds, the peripheral distribution persisted but was primarily concentrated in the southeastern and northwestern areas. Finally, in the fifth and sixth samplings, a pattern similar to the first sampling was observed, with a higher number of peripheral localities showing positive cases, although with a more marked impact in the southeastern and northwestern areas (Figure 2A). A predominance of positive cases is observed in peripheral localities, particularly in the southern, southeastern, and northwestern areas of the city with recurrent hotspots appearing across multiple sampling rounds.

Overall, most positive cases during the follow-up period were reported in the localities of Engativá and Puente Aranda, located in the northwestern and west-central areas of the city, respectively. Other localities exhibited greater variability in the number of positive cases (Figure 2A).

During the 6-month follow-up of the 109 enrolled subjects, 53 completed all six sampling time points and responded to the questionnaire. Figure 2B shows the individual data for total anti-N antibodies expressed as optical density (OD) values, which ranged from 0.147 to 4.004, and were considered positive when exceeding the established cutoff value. The data were not normally distributed (Shapiro–Wilk test, *p* < 0.001). Overall, 30.18% of participants (*n* = 16/53) showed SARS-CoV-2-specific anti-N antibodies, of whom 11.31% (*n* = 6/53) were women and 18.86% (*n* = 10/53) were men. Based on the estimated disease prevalence used for sample size calculation, the statistical power was 78% with an alpha level of 0.02. Using prevalence estimates reported in previous studies [36] for this population (age group 18–29 years) in Bogotá during 2020–2021, the statistical power increased to 99% with an alpha level of 0.0158.

#### 3.1.2. Attrition Bias Analysis

Within the cohort of 109 individuals enrolled at baseline between 20 and 29 years, 53 completed all six sampling rounds, whereas 56 discontinued follow-ups at different time points in the phase I (Figure 1 A,B). Table 1 summarizes the demographic, behavioral, and symptoms characteristics of the 53 completing participants, all proportions are presented with their exact 95% confidence intervals (CI) (Clopper-Pearson method).

To assess whether differential attrition could have biased the unmeasured study findings, baseline characteristics (sampling 1 (S1)) were compared between completers (*n* = 53) and dropouts (*n* = 56), and statistically significant differences were observed (*p* < 0.05) in antibody levels and seropositivity at baseline (Table 2). Participants who dropped out of the study had significantly higher baseline OD levels (mean 0.98 vs. 0.41, *p* = 0.0006) and a fourfold higher baseline seropositivity rate (26.8% vs. 5.7%, *p* = 0.0038) compared with those who completed follow-up. These findings suggest the possibility of differential attrition, which should be considered when interpreting seroconversion estimates.

#### 3.1.3. Risk Factors and Associated Signs and Symptoms

Based on the aforementioned findings, an analysis of risk factors and associated signs and symptoms was performed using a questionnaire from 88 participants with S1 and their corresponding antibody measurement.

**Figure 3:**
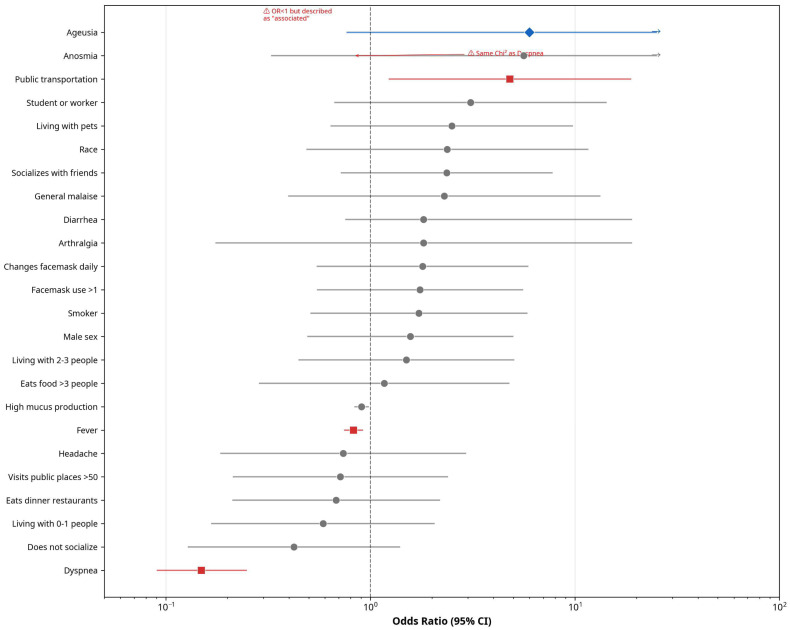
Forest Plot: Odds Ratios (95% CI) for SARS-CoV-2 Seropositivity (Table 3, Phase I). The red bars represent *p* < 0.05, the blue bars represent *p* < 0.10 and the gray bars represent *p* ≥ 0.10 bars.

The main risk factor associated with the presence of SARS-CoV-2 antibodies was the use of public transportation as the primary mode of mobility; however, the confidence intervals were wide, likely reflecting the limited sample size (Figure 3). Specifically, individuals who frequently used public transportation had a 4.8-fold higher probability of SARS-CoV-2 infection compared with those who used other means of transport. No statistical association was found for the remaining variables analyzed that would allow them to be identified as risk factors. Additionally, although men showed a higher probability of being seropositive compared with women, this difference was not statistically significant and therefore does not indicate a higher risk of infection in this group (Table 3).

With respect to signs and symptoms associated with antibody positivity, no statistical relationship was identified through OR estimation. However, the Pearson’s Chi-square test showed that increased mucus production, fever, and difficulty breathing were associated with seropositivity. Furthermore, Fisher’s Exact Test did not identify any additional variables associated with the presence of antibodies.

Regarding fever (OR = 0.827, 95% CI: 0.749–0.914) and dyspnea (OR = 0.149, 95% CI: 0.091–0.247), both were paradoxically and inversely associated with seropositivity, suggesting that these symptoms were more prevalent among seronegative individuals (Table 3 and Figure 3).

**Figure 4:**
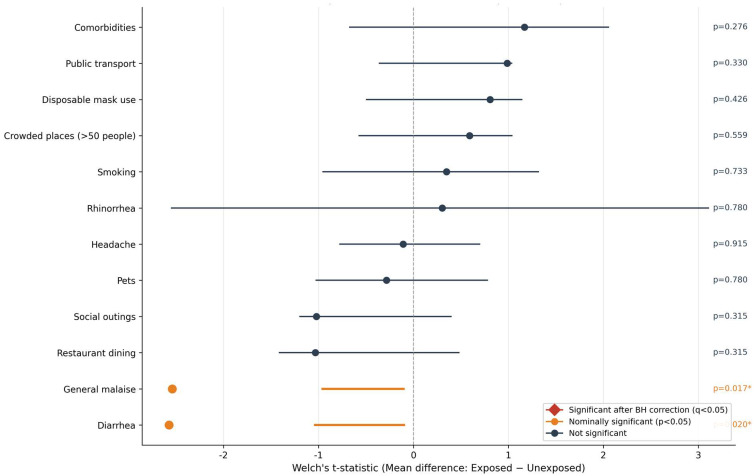
Association between risk factors and Total Ig OD for SARS-CoV-2 at month 6 (S6). Phase I completers, *n* = 53; Welch’s *t*-test; * *p* < 0.05; Red: significant after BH correction (q < 0.05); orange: nominal significant (*p* < 0.05); and black: not significant.

The Welch’s *t*-test was used to compare the mean OD value at sampling 6 (S6) between exposed (Yes) and unexposed (No) groups for each risk factor. ANOVA was used for categorical variables with more than two levels (marital status). The Benjamini–Hochberg correction (q-BH) was applied to control the false discovery rate (Table 4 and Figure 4). General malaise and diarrhea showed nominally significant associations (*p* = 0.017 and *p* = 0.023, respectively) with lower OD levels. However, the group sizes were very small (*n* = 6 and *n* = 4), and statistical significance was lost after correction for multiple comparisons (q-BH > 0.16); therefore, these findings should be considered exploratory. No other behavioral variables (smoking, living with pets, social activities, etc.) showed significant associations. None of the variables showed a statistically significant association with seropositivity (Supplementary Table S1 and Supplementary Figure S1).

#### 3.1.4. Functional Principal Component Analysis

This analysis allowed the identification of the first three principal components that explained the largest proportion of variability in the longitudinal antibody data. The first component (PC1) captured approximately 67.1% of the total variability and reflected differences in the magnitude and persistence of antibodies, distinguishing individuals with high and sustained responses from those with consistently low values. The second component (PC2) explaining 17% of the variability, primarily represented differences in the temporal shape of the antibody trajectories, including early-rise patterns, progressive declines, marked fluctuations, or relatively flat profiles.

**Figure 5:**
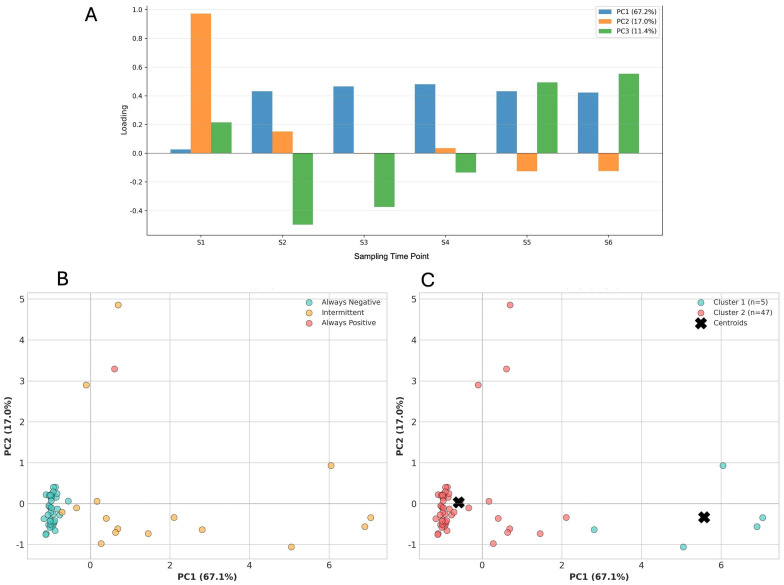
Functional Principal Component Analysis. (**A**) Uploads of the first three principal components (PC1-PC3). PC1 shows positive weights across all sampling points, reflecting overall antibody magnitude and persistence, while PC2 contrasts early and late sampling points, capturing differences in temporal antibody dynamics. PC3 reflects localized or transient peaks with limited contribution to overall variance. B and C. The projection of individual antibody trajectories onto the functional principal component space defined by PC1 and PC2. (**B**) Biplot by positivity pattern of PC1 (67.1% of explained variance) versus PC2 (17.0%), with points colored according to the qualitative serological positivity patterns identified during Phase I (“Always Negative,” “Intermittent,” and “Always Positive”). Individuals classified as “Always Negative” cluster predominantly on the left side of the plot, corresponding to low PC1 scores and consistently low antibody levels, whereas “Always Positive” individuals are mainly located on the right, reflecting higher PC1 values and sustained antibody responses. Intermittent patterns occupy intermediate and more dispersed regions of the PC space, indicating heterogeneous temporal dynamics. (**C**) K-means clustering (*k* = 2) performed on the same PC1–PC2 space. Points are colored by cluster assignment, and black markers indicate cluster centroids.

The third component (PC3) captured 11.4% additional variability associated with localized or transient increases in antibody levels, such as short-lived peaks observed in a subset of individuals (Figure 5A). Although PC3 contributed to describing these secondary features, it explained a substantially smaller proportion of the overall variance and did not improve the separation of immunological profiles or the interpretability of downstream analyses. Therefore, subsequent clustering, visualization, and survival analyses focused on PC1 and PC2, which together captured the dominant and biologically meaningful dimensions of antibody dynamics in this cohort.

The two-dimensional representation in the PC1–PC2 space revealed natural groupings that closely aligned with the initial qualitative classification, confirming that the curves, can be organized into coherent and reproducible immunological patterns (Figure 5B).

#### 3.1.5. Clustering Analysis Based on Principal Components

The clustering analysis, based on the first two principal components identified two distinct group within the cohort. The optimal number of clusters (*k* = 2) was determined using the Silhouette index. One cluster (cluster 1) comprised individuals with higher PC1 scores and a greater number of positive antibody measurements over time, whereas the second cluster (Cluster 2) included individuals with lower PC1 scores and fewer positive measurements throughout the follow-up period, consistent with the “always-negative” group (Figure 5C).

#### 3.1.6. Analysis of Risk Factor

The analysis of risk factor, based on the score of the first principal component (PC1), showed that most of the categorical variables evaluated did not present statistically significant associations, especially after applying the Benjamini–Hochberg correction for multiple comparisons (all q-values > 0.05) with antibody levels. The Independent Student’s *t*-tests were applied to variables with two categories, and the one-way ANOVA was used for variables with more than two levels, systematically comparing PC1 values across groups.

**Figure 6:**
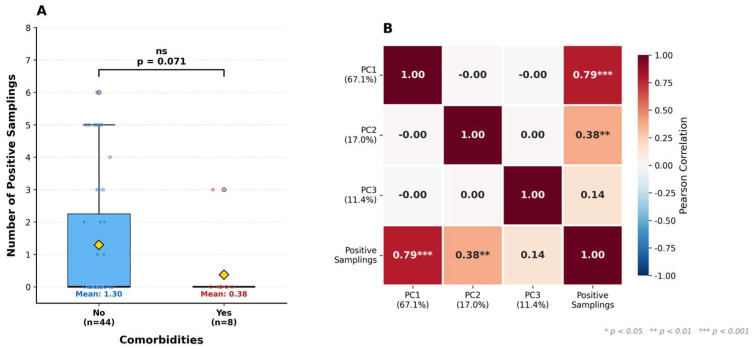
Analysis of the risk factor and correlation structure. (**A**) Distribution of the number of positive samplings by status of comorbidity. Individuals with comorbidities (*n* = 8) showed a lower mean number of positive samplings compared to those without comorbidities (*n* = 44), with a marginally significant trend (*p* = 0.071). Diamond markers indicate group means. (**B**) The Pearson correlation matrix between principal components (PC1, PC2, PC3) and the number of positive samplings. Significance levels: * *p* < 0.05, ** *p* < 0.01, *** *p* < 0.001. ns: not significant.

Among the categorical variables evaluated, comorbidities showed a marginally significant trend (Welch’s *t*-test, *p* = 0.071), with individuals reporting underlying health conditions exhibiting a lower mean number of positive samplings (0.38 ± 1.06) compared to those without comorbidities (1.30 ± 1.98). Although this association did not reach the conventional threshold of statistical significance (*p* < 0.05), the observed trend suggests that the presence of comorbidities may influence the frequency of seroconversion events. Furthermore, the Pearson correlation analysis revealed a strong, statistically significant positive correlation between the PC1 score (67.1% of variance) and the total number of positive samplings (r = 0.79, *p* < 0.001), confirming that the principal component of antibody dynamics is directly related to the frequency of positive results during the follow-up period, while PC2 shows a moderate correlation (r = 0.38, *p* < 0.01). (Figure 6).

#### 3.1.7. Predictive Analysis of Antibody Dynamics

Based on the knowledge gained during the pandemic, and to visualize and understand the longitudinal dynamics of antibody levels beyond the period of observation in young individuals, a predictive model was implemented. This approach provides valuable insight into the natural kinetics of antibody responses following infection and contributes to understanding the duration and behavior of humoral immunity in young adults under natural exposure conditions, prior to the introduction of COVID-19 vaccines in the country. Given the limited sample size, these projections should be interpreted as hypothesis-generating rather than definitive predictions.

The predictive analysis revealed differential patterns in the estimated evolution of antibody levels over twelve months, showing clear correspondence between the immunological behavior observed in Phase I and the trajectories projected by the model. The performance of the model was evaluated using a 5-fold cross-validation strategy with separation by individual, such that observations from the same participant were never simultaneously included in training and validation sets. Across folds, the Random Forest model achieved a mean absolute error (MAE) of 0.3788 ± 0.1010, a root mean squared error (RMSE) of 0.6768 ± 0.2528, and a coefficient of determination (R^2^) of 0.5635 ± 0.2968. The variability in R^2^ across folds likely reflects the limited sample size and high interindividual heterogeneity of antibody responses, consistent with the exploratory nature of this analysis.

**Figure 7:**
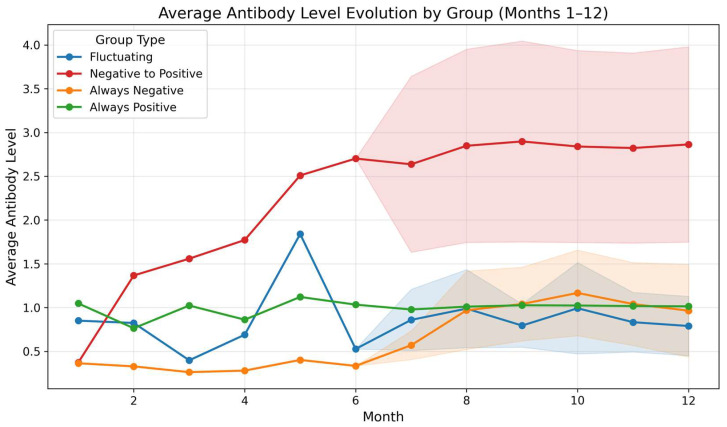
Predicted average trajectories of antibody levels from month 1 to month 12, grouped according to the serological patterns identified during Phase I. Solid lines represent the mean predicted antibody levels for each group, while shaded areas indicate the variability around the mean (prediction dispersion). The predictions were generated using a Random Forest model trained on longitudinal antibody measurements from the first six months and evaluated through 5-fold cross-validation with separation by individual.

Overall, the predictions reproduced the heterogeneity of individual dynamics and allowed the identification of well-defined group trends across the different serological categories. The negative-to-positive group (seroconversion) showed the most pronounced and sustained increase. In contrast, individuals who were always positive exhibited a more stable curve, with intermediate values and a slight upward trend toward the final months—consistent with persistent immune responses, but less abrupt than those observed in recent seroconversion cases. Meanwhile, the fluctuating group displayed the greatest intra-group variability, with irregular rises and falls reflected in wide dispersion intervals during months 8 to 12. Finally, the always-negative group showed persistently low levels throughout the entire period of evaluation, with no significant increases, indicating either a sustained absence of detectable response or insufficient immunological stimuli to generate measurable antibodies (Figure 7). These patterns provide an initial framework that could be substantially refined and validated in future studies with larger cohorts and extended follow-up periods.

### 3.2. Phase II

#### 3.2.1. Antibody Levels and Descriptive Analysis of the Immune Response

Phase II of the study was designed to evaluate the seroprevalence and persistence of SARS-CoV-2 antibodies in the same cohort of young adults following the implementation of the national COVID-19 vaccination program in Colombia (June 2021–December 2022) (Supplementary Tables). Thirty-six months later, a follow-up evaluation of the memory response was conducted in a subgroup of individuals (*n* = 29) from the 53 participants originally analyzed during the six-month follow-up of the pandemic.

**Figure 8:**
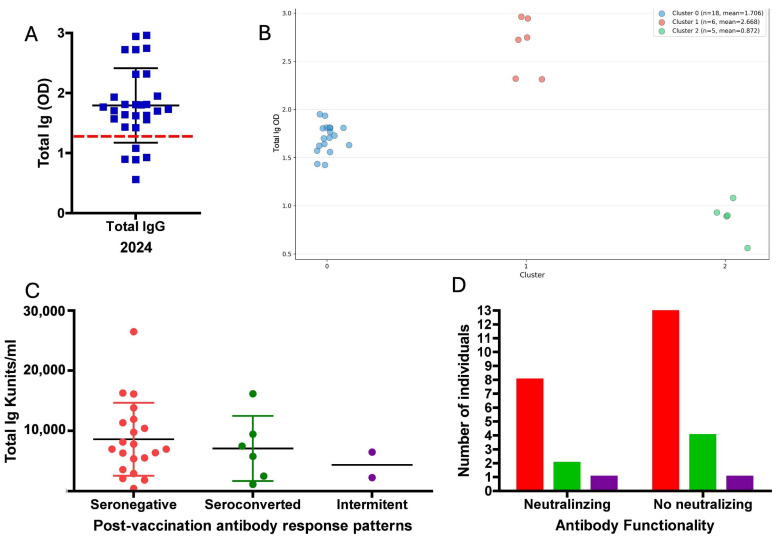
Total immunoglobulin (Total Ig OD) levels measured in 2024 among the Phase II cohort, distribution and K-means clustering (*k* = 3). (*n* = 29 subjects) (**A**) Individual data for total anti-S antibody OD values, each point represents the OD value for an individual. The red dotted line indicates the cutoff value (OD > 1.3). Data are presented as mean ± standard deviation. (**B**) Individuals are grouped into three distinct long-term immune response profiles based on their antibody levels (OD): a low-response cluster (Cluster 2), a medium-response cluster (Cluster 0), and a high-response cluster (Cluster 1). Each point represents an individual participant. (**C**) Post-vaccination antibody titers. Each point corresponds to Total Ig titers (Kunits/mL), distributed across groups of individuals according to their experience in Phase I. The median (IQR) for each group is shown. (**D**) Evaluation of the neutralizing capacity of IgG antibody titers after vaccination, the number of individuals testing positive in the functional (neutralization) assay for each group is shown. The red bars represent seronegative individuals, the green bars represent seroconverted individuals, and the purple bars represent intermittent responders.

Antibody levels (Total Ig OD) in 2024 showed a mean of 1.7929 with a standard deviation of 0.62 (Figure 8A). The data were normally distributed (Shapiro–Wilk test, *p* = 0.11667). SARS-CoV-2-specific anti-S antibodies were detected in 79.31% of participants (*n* = 23/29), of whom 47.82% (*n* = 11/23) were women and 52.17% (*n* = 12/23) were men. IgM antibodies were also quantified in all individuals, and only one subject tested positive.

#### 3.2.2. Clustering Analysis

The Silhouette Score analysis indicated that the most robust partition of the data corresponded to *k* = 3 clusters, with a score of 0.724. This suggests three descriptive groupings based on antibody levels, which may reflect distinct long-term immunological profiles: Cluster 0, composed of individuals with a medium response (*n* = 18; mean Total Ig OD = 1.706); Cluster 1, consisting of individuals with a high response (*n* = 6; mean Total Ig OD = 2.668); and Cluster 2, grouping individuals with a low response or indeterminate (*n* = 5; mean Total Ig OD = 0.872) (Figure 8B).

#### 3.2.3. Analysis of Post-Vaccination Antibody Titers

With the aim of evaluating the vaccine induced memory response in this cohort and analyzing its relationship with the natural history of the disease observed during phase I, particularly in the context of hybrid immunity generated by the combination of natural infection and vaccination [37,38], antibody titers (Kunits/mL) obtained from the 29 subjects after vaccination (Supplementary Tables) were examined.

The data were grouped according to the subject’s response profiles, based on their distribution within the principal components (PC1 and PC2) identified in phase I, together with the previously defined qualitative seropositivity patterns, classified as “always negative” (seronegative), “intermittent”, and “always positive”, the latter corresponding to the seroconverted group (Figure 8C,D).

Of the participants, 79.31% (23/29) had positive antibody titers, 17.24% (5/23) exhibited antibody titers associated with prior infection plus vaccination, whereas 62.07% (18/23) of the vaccinated participants without prior infection also showed antibody titers.

Based on antibody prevalence percentages reported in previous studies [39] for individuals over 18 years of age in Colombia, and considering participants recruited between March and June 2024, the statistical power was 71.30% with an alpha level of 0.02.

The data did not follow a normal distribution according to the Shapiro–Wilk test (*p* = 0.011). No significant differences were found in antibody titers based on the immune behavior observed in the first phase or on the history of natural COVID-19 infection (*p* = 0.265) (Figure 8C). Additionally, the study evaluated whether the vaccine-induced antibodies, two years after immunization, retained neutralizing or functional capacity [40]. A binomial test showed no significant differences (*p* = 0.20); however, this finding should be interpreted with caution. Given the small sample size (*n* = 29) and the very low observed frequency of neutralizing antibodies, the study was substantially underpowered to detect anything other than very large effects. Accordingly, the non-significant *p*-value should not be interpreted as evidence for the absence of neutralizing capacity, but rather as reflecting the low frequency of detectable neutralization in this cohort under the conditions evaluated (Figure 8D).

#### 3.2.4. Analysis of Risk Factor

*p*-values were calculated using Welch’s *t*-test to compare the mean OD values in 2024 between the exposed and unexposed groups for binary variables and ANOVA for multi-group variables. The Benjamini–Hochberg correction (q-BH) was applied.

Regarding the analysis of risk factors for categorical variables, most factors evaluated (including having pets, marital status, smoking, and comorbidities) showed no statistically significant association with total Ig levels (OD) after the normalization of data.

However, two variables of interest emerged. A Welch’s *t*-test revealed a statistically significant difference based on prior COVID-19 infection (t(27) = −2.214, *p* = 0.036). Paradoxically, individuals who had not previously contracted COVID-19 exhibited higher mean antibody levels (OD = 2.034, *n* = 11) compared to those who had been infected (OD = 1.595, *n* = 18). This may suggest a more robust or recent response to vaccination in the uninfected group. Additionally, a marginally significant trend was observed for teleworking (t(27) = −1.920, *p* = 0.066), where individuals not working from home tended to have higher antibody levels. The complete statistical results for these categorical variables are presented in Table 5.

**Figure 9:**
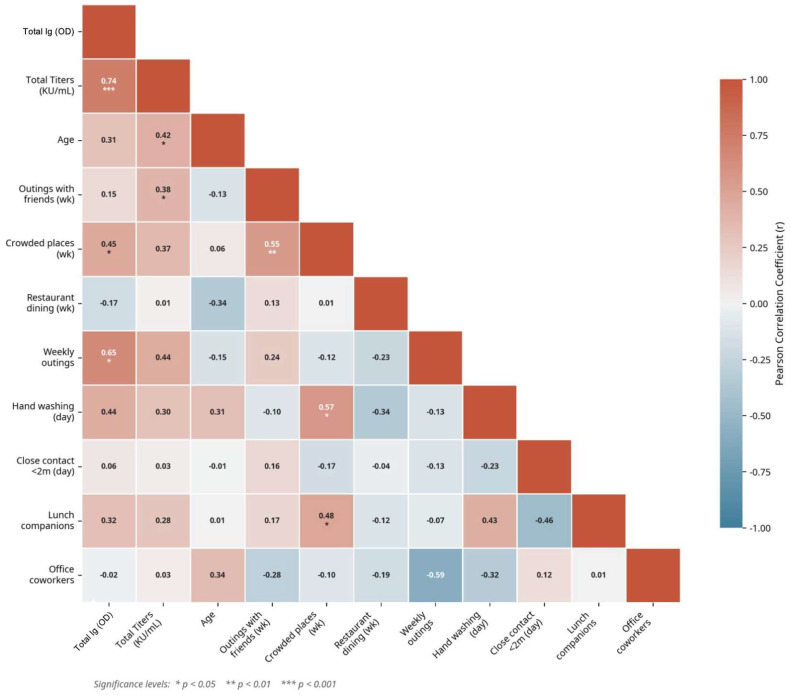
Correlation Matrix of Total Ig (OD) and Social Behavior Variables. The matrix displays Pearson correlation coefficients (r) between key numerical variables. Statistically significant correlations are marked with asterisks (* *p* < 0.05, ** *p* < 0.01, *** *p* < 0.001). A significant positive correlation is observed between Total Ig (OD) and both ‘Crowded places’ (r = 0.45) and ‘Weekly outings’ (r = 0.65), and a marginally significant trend is noted with ‘Hand washing’ (r = 0.44, *p* = 0.076).

In the analysis of numerical variables, a correlation matrix was constructed to explore associations between antibody levels and social behaviors. Contrary to initial assumptions, dining at restaurants showed no significant correlation. Instead, the analysis revealed that the frequency of exposure to social settings was significantly related to antibody levels. As shown in Figure 9, a moderate and significant positive correlation was found between Total Ig levels (OD) and the frequency of visiting crowded places (r = 0.45, *p* = 0.013). A stronger positive correlation was also observed with the general frequency of weekly outings (r = 0.65, *p* = 0.042).

## 4. Discussion

In this study, antibody levels against SARS-CoV-2 were evaluated in a group of young adults aged 20 to 29 years residing in the city of Bogotá, Colombia, during the pandemic (2020 to 2021) in an initial phase. Subsequently, a second phase was conducted following the national vaccination program (June 2021–December 2022), including a subgroup of participants from the initial phase who were evaluated 36 months after the last blood draw of the first phase and two years post-vaccination. This design enabled the integration of early-pandemic serological profiles resulting from natural infection with a long-term post-vaccination assessment in the same individuals, providing a novel insight into the role this population played in dissemination of the virus during the pandemic and in immune memory in young adults after vaccination [26,27].

While antibody kinetics and waning immunity to SARS-CoV-2 have been extensively characterized, the contribution of this study lies in its contextual and population-specific perspective. By focusing on young adults aged 20–29 years in Bogotá, this work addresses a demographic that plays a central role in viral transmission; however, it remains unrepresented in longitudinal immunological studies, particularly in low- and middle-income countries [41].

Importantly, the longitudinal design identified the antibody dynamics during an epidemiological period with intense community transmission and the circulation of the Mu (B.1.621) variant, a lineage first identified in Colombia [42]. Although individual-level variant confirmation was not available, the temporal overlap between follow-up and Mu dominance provides relevant epidemiological context for interpreting the observed antibody dynamics.

A total of 109 subjects were included, with a seroprevalence of 15.59% for antibodies against the SARS-CoV-2 N protein. Among those included in the complete analysis of Phase I (*n* = 53), a seroconversion probability of 30.18% was observed during the follow-up period (December 2020 to May 2021). The highest number of cases were concentrated in the southern, northwestern, and central-western areas of Bogotá, and frequent use of public transportation was determined as the main risk factor. On the other hand, high mucus production, fever, and respiratory difficulty were the most representative signs/symptoms of infection, consistent with previous reports [23,43,44,45,46,47] although many individuals were asymptomatic [12,13,48,49].

Even though, anti-N antibodies have been shown to decrease in some cohorts [50], and the neutralizing capacity of these antibodies was not determined in this study, it has been widely shown that the levels of antigen binding antibodies are correlated with disease progression and symptomatic infections appearance [51,52,53]. Therefore, the description of the anti-N antibodies levels in our cohort, along with other studies, contribute to a framework for preparedness against future outbreaks, and reinforces their utility as markers of infection.

Regarding local (Bogotá) and national reports issued by the National Health Observatory, a higher number of active daily infection cases and a greater percentage of positive tests were detected between January-February and April-May for all age groups during the follow-up period from December 2020 to May 2021 [54,55]. This pattern correlates with the monthly incidence behavior detected during the follow-up period and could be explained by the infection peaks reported in Colombia for December–January 2020 and May–June 2021 due to an increase in the transmission rate [56]. However, Colombia reported a cumulative incidence (CI) of 3–6% for all age groups during the same follow-up period, which is considerably lower than the values observed in this study [57].

This discrepancy may suggest that populations with specific characteristics such as age—individuals aged 20 to 29 years—and/or city of residence—inhabitants of Bogotá—may exhibit a higher probability of infection compared to the national average and with the expected behavior in other age groups and cities.

At a district level, the highest number of active cases across all age groups was reported in the localities of Suba (84,965 cases), Kennedy (71,448 cases), Engativá (64,810 cases), Usaquén (46,861 cases), Bosa (37,602 cases), Fontibón (31,151 cases), and Ciudad Bolívar (28,028 cases), corresponding to the northern, southwestern, northwestern, western, and southern areas of the city [58]. These data differ slightly from the spatial distribution of seropositive cases identified in this study, likely because only individuals from a specific age group were included and because the number of participants per locality was not homogeneous. Additionally, only individuals from 17 of the 20 localities that comprise the urban area of Bogotá were sampled.

In our cohort, four distinct patterns of anti-N antibody dynamics were identified among seropositive individuals; the majority showed seroconversion with high antibody levels within 1–2 months post-infection (mpi), followed by a slightly decline, with detectable antibodies persisting in some cases up to 4 mpi. The detection of anti-N antibodies allowed us to describe the epidemiological dynamics of infection and reinfection in this group of young adults during the pandemic. Notably, most infections and reinfections occurred during the fifth and sixth months of follow-up, aligning with documented transmission and reinfection peaks in several countries, including epidemiological evidence in Colombia [59,60].

Consistent with our observations, several longitudinal studies have reported heterogeneous patterns of anti-N antibody responses, with most individuals seroconverting and reaching peak titers within the first 1–2 weeks to several months post-infection, followed by a progressive decline over 3–6 months. However, in some individuals, anti-N antibodies remain detectable for 4 months or longer [59,61,62]. Consistent with these findings, it has been described that anti-N and anti-S antibody production begins around 12 days post-infection (dpi), with an average duration of up to 12.5 months post-infection (mpi), where approximately 82% of cases show a gradual decrease in antibody levels, while 13.1% maintain stable titers throughout follow-up [63,64,65]. It is important to note that it has been described elsewhere that anti-N antibodies can vary significantly depending on the time post-infection severity of the disease and vaccination. Furthermore, compared to anti-Spike antibodies, anti-N antibodies decrease more rapidly over time, which in some cases it has been reported that almost 40% of individuals lose seropositivity. The shorter average duration observed in this study could be explained by these previous reports.

Despite this variability, other studies have reported that antibodies can persist between 5 and 8 months in infected individuals, depending on the antigen used for detection and the clinical severity of the condition, which reinforces the idea that there is no uniform or standardized humoral response against SARS-CoV-2 [64]. On the other hand, in a research study conducted in a native population of the Colombian Amazon Region, it was determined that there is no statistically significant difference in antibody dynamics with respect to age [66]. In this context, the high variability observed in antibody dynamics in this study is consistent with that previous report across diverse populations and could be extrapolated to other age groups.

After conducting follow-up of the study population and measuring anti-N antibody levels against SARS-CoV-2, the first phase revealed that the main risk factor for acquiring the infection was the use of public transportation as a means of transport (OR 4.813; 95% CI 1.23–18.69). This finding is consistent with the Latin American context, where public transportation constitutes the primary mode of mobility [67] as well as with the characteristics of overcrowding and close contact that favor aerosol and fomite transmission in crowded transport systems such as those in Bogotá [68]. The presence of the virus on surfaces and in public transportation environments has been demonstrated in various studies [69,70,71], and similar results regarding increased risk of infection associated with the use of public transit have been reported in Norway [72], China [73], and the United States [74].

These exposure patterns are also reflected in the variability of anti-N antibody kinetics observed in this study and other studies. Frequent use of public transportation may promote continuous re-exposure, as these settings facilitate viral transmission. Evidence from Sweden, where over 50% of public transportation workers were seropositivity for SARS-CoV-2, supports this notion [74]. Additionally, factors such as exposure time, ventilation, and passenger density have been shown to increase the risk of transmission [75]. Together, these findings suggest that the serological dynamics observed in the cohort likely reflect a balance between the natural decline of IgG and repeated re-exposure driven by social mobility and the use of public transportation.

Regarding fever (OR = 0.827, 95% CI: 0.749–0.914) and dyspnea (OR = 0.149, 95% CI: 0.091–0.247), both showed an inverse association with seropositivity, suggesting a higher observed frequency of these symptoms among seronegative individuals. This finding may reflect a behavioral effect, whereby participants experiencing more severe symptoms may have self-isolated more strictly, thereby reducing their exposure to the virus. Given the small number of symptomatic cases for these variables, the corresponding effect estimates may be imprecise and should be interpreted accordingly.

Beyond these observed patterns, a critical observational gap remained between the completion of the six-month serological follow-up and the subsequent implementation of the national vaccination program in this age group, during which dynamics could not be directly assessed. To address this limitation, we developed a predictive model based on the longitudinal data obtained during Phase I.

By applying a machine learning approach to time series data, the model extended antibody trajectories from the first six months up to twelve month, while preserving interindividual heterogeneity in immune responses [76,77]. Rather than generating individual-level clinical predictions, this approach captured average patterns and group-level trends consistent with the serological profiles defined in Phase I, providing an integrated representation of immunological behavior over time.

However, these findings should be interpreted cautiously given methodological constraints, including the small sample size and the recursive forecasting structure, which may have contributed to variability across cross-validation folds. Accordingly, the model should be considered an exploratory tool for identifying general longitudinal patterns. Future studies with larger and more diverse cohorts, along with external validation, are needed to improve robustness and generalizability.

At both district and national levels, these findings provide a useful framework for understanding the role of young adults in transmission dynamics and for guiding targeted prevention and control strategies. From a public health perspective, they also reinforce risk perception and highlight the importance of vaccination, which not only protects individuals but also contributes to collective immunity and has been essential in reducing transmission of SARS-CoV-2 and the severity of the disease [78]. In this context, the availability of predictive tools such as the one proposed here could help reduce uncertainty in future outbreaks, supporting more informed public health and clinical decision-making. This is particularly relevant given that, during the early stages of the pandemic, limited knowledge of disease progression contributed to the inappropriate use of antibiotics in both community [79,80], and healthcare settings, ultimately exacerbating antimicrobial resistance [81,82,83]. Together, these findings underscore the broader value of integrating epidemiological insights with predictive modeling to optimize intervention strategies and improve health system response.

In Colombia, the national COVID-19 vaccination program began in February 2021 under a phased prioritization strategy targeting healthcare workers, older adults, and individuals with comorbidities [84,85,86]. Young adults (20–29 years) were incorporated in later stages of the rollout (Phases 4 and 5, June–July 2021) making them among the last group to be vaccinated [87,88]. This occurred during a period of intense community transmission and circulation of multiple SARS-CoV-2 variants in Colombia (Gamma, Mu, and Delta) in urban centers such as Bogotá [89]. It is important to note that individual-level viral genomic sequencing was not available for participants in this study. Consequently, variant-specific attribution at individual level could not be performed. The epidemiological context of variant circulation was inferred based on contemporaneous national genomic surveillance data from Colombia, which documented the dominance of the Mu (B.1.621) variant during the main follow-up period (December 2020 to May 2021). All variant-related interpretations in this study should be understood at the population level rather than as direct individual-level associations [90].

Nationwide, including mRNA vaccines (BNT162b2 and mRNA-1273), viral vector vaccines (ChAdOx1nCoV-19 and Ad26.COV2.S), and inactivated virus vaccines (CoronaVac) (Supplementary Table S2), contributed to heterogeneous immune response profiles at the population level [91]. Given the high risk of exposure, mobility, and ongoing viral transmission during vaccination rollout, many individuals likely developed hybrid immunity through the combined effects of natural infection and vaccination, a phenomenon increasingly recognized in Colombian cohorts and global immunological studies [39,92].

This epidemiological and immunological context provides a relevant framework for interpreting antibody kinetics, neutralizing capacity, and the durability of the immune response. Within this framework, Phase II of this study was conducted to evaluate long-term immunological memory in young adults in Colombia.

Accordingly, most individuals in this small cohort remained seropositive for total antibodies two years after vaccination. Prior natural exposure to the antigen (phase I) did not confer higher post-vaccination antibody titers. Regarding neutralizing capacity, the low frequency of detectable neutralizing antibodies observed in phase II, combined with the limited sample size (*n* = 29), precludes definitive conclusions about the presence or absence of a neutralizing response. These results should be considered preliminary and hypothesis-generating, warranting confirmation in larger, adequately powered studies. In line with previous reports, these findings suggests that the magnitude of the initial immune response does not necessarily predict long-term antibody levels several years later and may have implications for viral dissemination in the post-pandemic context, considering that SARS-CoV-2 has now acquired an endemic status [90].

Furthermore, the magnitude and duration of antibody levels showed considerable variability, which may indicate reinfection or immune memory response. This is consistent with previous evidence, as various studies have demonstrated that protective anti-SARS-CoV-2 antibodies induced by vaccines decline gradually over time: for example, six months after the second dose of BNT162b2, a substantial decrease in humoral response is observed [93], and other studies report a decline in anti-spike antibody titers at 5–6 months post- vaccination [94]. Furthermore, it has been previously described that neutralizing IgG levels decrease over time and that cross-reactive immune protection against SARS-CoV-2 variants depends on sequential antigen exposure either by vaccination, natural infection, or both (hybrid immune response) [95]. In fact, we observed one individual that tested positive for IgM in phase II, indicating re-infection despite vaccination or previous natural infection. Thus, this result observed in our study reinforces the idea that subsequent antigen exposure generates protective neutralizing antibodies against variants, highlighting the need for booster doses, which have been recommended to maintain adequate protection [96].

Additionally, the emergence of variants in Colombia and in several countries worldwide decreased the ability of vaccines to confer protection against reinfection [96], a pattern that was also observed in the present study.

Furthermore, the emergence of viral variants has decreased the ability of vaccines to confer protection against reinfection; multiple lineages have demonstrated escape capacity from antibody neutralization [97], including variants carrying Spike protein mutations that affect immunity efficacy [42]. In Colombia, for example, the Mu (B.1.621) variant has been identified for its possible resistance to vaccine-induced neutralizing antibodies [89], which is consistent with the patterns observed in this study. Our results, although exploratory, in line with previous studies and academic reviews [78], highlight the importance of vaccination as a prevention strategy for SARS-CoV-2 transmission, but also as a pandemic preparedness strategy for future outbreaks.

In this phase II analysis, prior SARS-CoV-2 infection was unexpectedly associated with lower antibody levels compared with infection naïve individuals, challenging the conventional assumption that hybrid immunity universally results in stronger humoral responses. While hybrid immunity has been shown to enhance both the magnitude of antibodies and durability in many settings [98,99], emerging evidence suggests that antibody dynamics may vary depending on the timing of infection, antigenic imprinting, and subsequent antigen exposure [100]. It is possible that individuals without documented prior infection experienced more recent antigenic stimulation through vaccination or unrecognized asymptomatic exposures, which have been shown to boost humoral responses and sustain antibody levels over time [101].

Additionally, marginally significant trend association was observed with teleworking, whereby individuals not engaged in teleworking tended to have higher antibody levels, supporting the hypothesis that increased environmental and occupational exposure may contribute to ongoing immune stimulation. Consistent with this interpretation, epidemiological studies have demonstrated that occupational and community exposure are independently associated with higher seroprevalence and sustained antibody responses [102].

Notably, we observed significant positive correlations between antibody levels and the frequency of exposure to social environments, including crowded places and general weekly outings. These findings align with previous reports demonstrating that repeated antigenic exposures, including subclinical or asymptomatic encounters, contribute to continued immune activation, affinity maturation of memory B cells, and the maintenance of circulating antibodies over extended periods [99,103].

Collectively, these results suggest that behavioral and exposure related factors, including the status of teleworking and the frequency of social interaction, may significantly influence long-term humoral immune dynamics, particularly in young adults living in environments with ongoing viral circulation.

This study has several important limitations that should be considered when interpreting the findings. First, participants were recruited through a non-probabilistic convenience sample strategy, which precludes any claim of statistical representativeness of the 20–29-year-old population of Bogotá and limits the generalizability of the results.

Second, the study’s statistical power is constrained by its sample size and the observed participant attrition, factors that necessitate a cautious interpretation of the findings, particularly in Phase II (*n* = 29), where the small sample size limits the ability to detect moderate or small effects. Accordingly, all results should be interpreted within their exploratory scope, and the possibility of multiple natural exposures over time should be considered when evaluating longitudinal antibody dynamics. Third, differences in baseline seropositivity between completers and dropouts suggest the possibility of attrition bias, which may have influenced seroconversion estimates. In addition, participant attrition was observed during follow-up, possibly due to reduced motivation or because, during the pandemic, many students returned to their homes as classes shifted to remote formats. This phenomenon, known as self-selection or differential participation bias, should be considered in the interpretation of the results and represents an important consideration for future serological studies.

Fourth, the observed seroprevalence differed from the value assumed in the calculation of the sample size, which may affect the precision of the estimates. Fifth, given the number of exploratory analyses performed relative to the sample size, findings should be interpreted as hypothesis-generating rather than confirmatory. Sixth, the analysis of clustering was based on serological data, and the profiles identified should be further validated using independent baseline covariates in future studies. Seventh, predictive modeling results showed variability across validation folds, likely reflecting sample size constraints and biological heterogeneity; accordingly, projected trajectories beyond the observation period should be interpreted with caution. Despite these limitations, this study provides an initial approach to understanding the epidemiological behavior of SARS-CoV-2 in young adults (20–29 years), based on data collected during the peak of the pandemic and considering both natural infection and vaccination.

In line with this, the predictive model should be regarded as an exploratory and hypothesis-generating tool rather than a source of individual-level prediction. Its main value lies in supporting future studies with larger cohorts, more robust longitudinal designs, and external validation (105).

While exploratory, these findings may offer contextually relevant insights for populations with similar biological and socioeconomic characteristics and contribute to understanding the role of young adults in transmission dynamics. At district and national levels, they may help inform targeted prevention, control, and mitigation strategies, particularly in the context of future outbreaks involving pathogens with similar transmission patterns.

## 5. Conclusions

This longitudinal exploratory observational study provides insights into the serological dynamics of SARS-CoV-2 during the pandemic and post-pandemic stages among young adults in Bogotá. By emphasizing the importance of epidemiological context when interpreting immune trajectories and long-term immune memory, it aims to contribute to a more nuanced understanding of this population’s potential role in viral epidemiology and to offer contextually relevant insights for public health strategies.

Regarding serological dynamics, infected individuals exhibited substantial variability, which precluded the definition of a generalized model and suggests that such approaches may be more appropriate for subgroups with similar response patterns. Accordingly, future seroepidemiological studies may benefit from further characterization of antibody responses to different viral proteins, particularly in the context of vaccine evaluation.

In Phase II, antibody levels allowed the identification of three long-term response clusters (low, medium, and high), with no clear association with early response observed in Phase I. While exploratory, these findings suggest that the initial magnitude of antibodies may not reliably predict longer-term immune response, highlights the complexity of humoral immunity.

Rather than proposing generalizable immunological models, these findings provide an exploratory description of how antibody kinetics and post-vaccination immune memory may evolve in a high-mobility urban population exposed during a period of variant-driven transmission. As such, they may offer insights that could inform public health surveillance and pandemic preparedness.

## Figures and Tables

**Table 1:** Baseline Characteristics of Study Completers.

Variable	*n*	Prevalence (%)	95% CI
Demographic			
Sex (% Female)	35	66.0%	[51.7–78.5%]
Age (mean ± SD)	53	24.8 ± 4.9%	[23.5–26.2%]
Behavioral			
Living with Pets	26	49.1%	[35.1–63.1%]
Smoker	10	18.9%	[9.4–32%]
Comorbidities	8	15.1%	[6.8–27.6%]
Socializes with friends	18	34%	[21.5–48.3%]
Visits public places with >50 people	24	45.3%	[31.6–59.6%]
Eats dinner in bars or restaurants	24	45.3%	[31.6–59.6%]
Symptoms			
Headache	15	28.3%	[16.8–42.3%]
General malaise	5	9.4%	[3.1–20.7%]
High mucus production	4	7.5%	[2.1–18.2%]
Diarrhea	4	7.5%	[2.1–18.2%]
Anosmia	2	3.8%	[0.5–13%]
Ageusia	1	1.9%	[0.0–10.1%]
Fever	1	1.9%	[0.0–10.1%]
Dyspnea	1	1.9%	[0.0–10.1%]

**Table 2:** Baseline Characteristics of Study Completers vs. Dropouts.

Baseline Characteristics (S1)	Completers (*n* = 53)	Dropouts (*n* = 56)	Test	*p*-Value
Mean OD (±SD)	0.41 ± 0.19	0.98 ± 1.15	Welch’s *t*-test	0.0006 *
Seropositivity (%)	5.7% (3/53)	26.8% (15/56)	Fisher’s Exact	0.0038 *

* *p*-value ≤ 0.05 indicates statistical significance.

**Table 3:** Association between risk factors, associated signs and symptoms and the presence of SARS-CoV-2 antibodies in (*n* = 88).

Variable	OR	CI Lower	CI Upper	Pearson’s Chi-Square	*p*-Value *
Personal information					
Male sex	1.569	0.495	4.969	0.591605	0.442
Race	2.377	0.49	11.541	1.211121	0.271
Activities					
Use of public transportation as the main mode of transportation	4.813	1.239	18.693	5.880	0.015 *
Living with 0–1 people	0.587	0.168	2.046	0.711367	0.399
Living with 2–3 people	1.500	0.448	5.018	0.436845	0.509
Living with pets	2.500	0.643	9.724	1.834749	0.176
Smoker	1.728	0.513	5.827	0.791126	0.374
Student or worker	3.091	0.672	14.207	2.275158	0.131
Eats food in a space with more than 3 people	1.169	0.287	4.754	0.047573	0.827
Socializes with friends after work or on weekends	2.363	0.722	7.735	2.094856	0.148
Does not socialize with friends on weekdays or weekends	0.423	0.129	1.386	2.094856	0.148
Eats dinner in bars or restaurants	0.680	0.213	2.176	0.424758	0.515
Eats dinner in bars/restaurants 1–3 times per week	0.839	0.765	0.920	0.191	0.6621 ^1^
Visits public places with >50 people	0.713	0.214	2.378	0.304358	0.158
Visits public places >1 time per week	0.833	0.757	0.916	0.792	0.3731 ^1^
Signs, symptoms, and preventive measures					
High production of mucus	0.905	0.841	0.975	1.438	0.230
Fever	0.827	0.749	0.914	5.346	0.021 *
Dyspnea	0.149	0.091	0.247	5.346	0.021 *
Headache	0.736	0.186	2.915	3.640375	0.662
Ageusia	6.00	0.77	46.749	1.777858	0.056
Anosmia	5.615	0.33	95.528	1.777858	0.182
General malaise	2.3	0.399	13.244	0.911442	0.34
Arthralgia	1.821	0.176	18.883	0.258871	0.611
Diarrhea	1.821	0.760	18.883	0.258871	0.611
Medical history (diabetes, obesity, gastric ulcers, cancer, liver or kidney disease)	0.835	0.760	0.918	0.587599	0.4431 ^1^
Other diseases	0.361	0.043	3.008	0.956336	0.328
Use of Facemask	0.604	0.19	1.913	0.745	0.388
Uses facemask more than once	1.75	0.552	5.55	0.917375	0.388
Changes facemask at least once	1.194	1.088	1.311	0.387178	0.5341 ^1^
Changes facemask daily	1.8	0.551	5.884	0.963029	0.326

* *p*-value ≤ 0.05 indicates statistical significance. ^1^ Two-sided Fisher’s Exact Test = 1.000. CI: Confidence Interval.

**Table 4:** Association between risk factors, associated signs and symptoms and the presence of SARS-CoV-2 antibodies (OD) in *n* = 53, Phase I.

Variable	*n* (Yes)	Median OD (Yes)	*n* (No)	Median OD (No)	t	*p*-Value	q-BH
Personal Information							
Marital status (ANOVA)	-	-	-	-	F = 0.35	0.705	0.814
Activities							
Social activities	18	0.828	19	0.941	−0.999	0.315	0.540
Restaurants	24	0.838	13	0.942	−0.999	0.315	0.540
Use of public transportation as the main mode of transportation	21	0.931	31	0.840	0.988	0.330	0.540
Visits public places with >50 people	23	0.908	14	0.835	0.589	0.559	0.745
Smoker	10	0.846	27	0.897	−0.344	0.733	0.814
Living with pets	25	0.855	12	0.923	−0.280	0.780	0.840
Signs, symptoms, and preventive measures							
General malaise	6	0.543	47	0.960	−2.394	0.017 *	0.162
Diarrhea	4	0.467	49	0.937	−2.580	0.023 *	0.162
Previous COVID-19 infection	1	0.811	36	0.887	−1.100	0.270	0.540
High mucus production	4	0.820	48	0.892	−0.280	0.780	0.840
Headache	14	0.862	38	0.893	−0.112	0.915	0.915
Use of facemask	17	0.943	20	0.830	0.799	0.426	0.639
Comorbidities	8	0.952	44	0.871	0.348	0.276	0.540

* *p*-value ≤ 0.05 indicates statistical significance.

**Table 5:** Association between the presence of SARS-CoV-2 antibodies and independent variables in the study population in Phase 2 (*n* = 29).

Variable	*n* (Yes)	Median OD (Yes)	*n* (No)	Median OD (No)	t	*p*-Value	q-BH	[CI 95%]
Personal Information								
Marital status ^+^	-	-	-	-	F = 0.818	0.453	0.532	-
Activities								
Living with pets	16	1.636	13	1.916	−1.260	0.220	0.384	−0.279 [−0.736, 0.178]
Smoker	3	1.504	26	1.791	−0.871	0.456	0.532	−0.287 [−1.422, 0.848]
Telework	9	1.520	20	1.870	−1.920	0.066	0.197	−0.350 [−0.725, 0.024]
Daily outings	24	1.809	5	1.535	1.330	0.213	0.384	0.273 [−0.185, 0.732]
Signs, symptoms, and preventive measures								
Previous COVID	18	1.595	11	2.034	−2.214	0.036 *	0.197	−0.438 [−0.845, −0.032]
Comorbidities	9	1.695	20	1.791	−0.419	0.681	0.681	−0.096 [−0.579, 0.387]

* *p*-value ≤ 0.05 indicates statistical significance. ^+^ ANOVA.

## Data Availability

The data supporting the findings of this study are stored in Excel spreadsheets on institutional and personal computers of the co-investigators. These data were collected through questionnaires completed by participants who provided informed consent prior to inclusion in the study. Due to privacy and ethical restrictions, the datasets are not publicly available. However, anonymized data may be provided by the corresponding author upon reasonable request.

## Supplementary Materials

The following supporting information can be downloaded at: https://www.mdpi.com/article/10.3390/biomedicines14040849/s1, Figure S1: Odds Ratios for Seropositivity at Month 6 by risk factors; Table S1: Risk Factors vs. Seropositivity (*n* = 53, OR); Table S2: Vaccination Details for Phase II Participants.
