# Supplementary material for: A Longitudinal Exploratory Study of SARS-CoV-2 Antibody Dynamics in Young Adults in Bogotá: Lessons from Natural Infection and Post-Vaccination Memory
Source: Biomedicines. 2026 Apr 8;14(4):849. doi: 10.3390/biomedicines14040849 (PMC13113533; doi:10.3390/biomedicines14040849)
Supplement: Supplementary file 1 [file biomedicines-14-00849-s001.zip › biomedicines-4200777-supplementary.pdf]

Supplementary Materials

Table S1: Risk Factors vs. Seropositivity (n = 53, OR)

Variable	E+/S+	E+/S-	E-/S+	E-/S-	OR	CI 95%	p-value
Activities							
Smoker	2	8	8	19	0.594	[0.103-3.437]	0.694
Living with pets	6	19	4	8	0.632	[0.139-2.862]	0.696
Visits public places with > 50 people	7	16	3	11	1.604	[0.339-7.597]	0.710
Social activities	4	14	6	13	0.619	[0.142-2.701]	0.714
Restaurants	6	18	4	9	0.750	[0.168-3.351]	0.716
Use of public transportation	6	15	8	23	1.150	[0.332-3.984]	1.000
Signs, symptoms, and preventive measures							
Dyspnea	1	0	13	38	ND	ND	0.269
Anosmia	1	1	13	37	2.846	[0.166-48.859]	0.470
Ageusia	1	0	13	38	ND	ND	0.269
Headache	4	10	10	28	1.120	[0.286-4.390]	1.000
General malaise	1	4	13	34	0.654	[0.067-6.409]	1.000
Diarrhea	1	3	13	35	0.897	[0.085-9.420]	1.000
High mucus production	1	3	13	35	0.897	[0.085-9.420]	1.000
Fever	1	0	13	38	ND	ND	0.269
Previous COVID-19 infection	1	0	9	27	ND	ND	0.270
Use of Facemask	6	11	4	16	2.182	[0.497-9.583]	0.460
Comorbidities	3	5	11	33	1.800	[0.369-8.789]	0.666

E: Exposure

S: Seropositivity

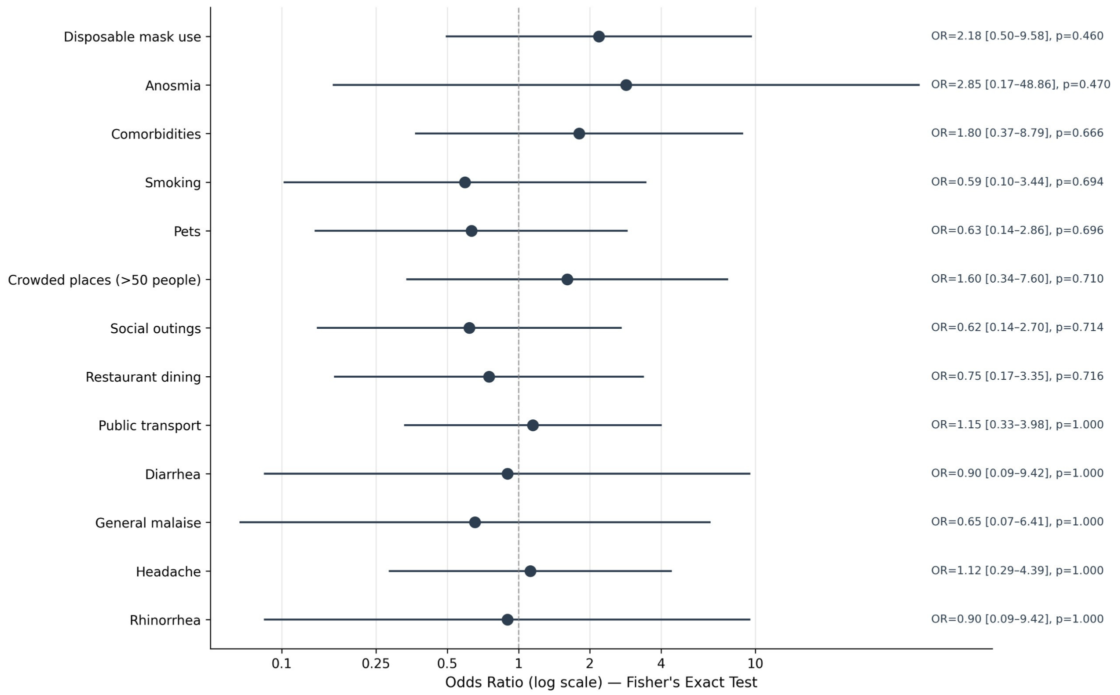

Figure S1. Odds Ratios for Seropositivity at Month 6 by risk factors. Phase I completers, n=53; Fisher's Exact Test; *p<0.05; ♦ q-BH<0.05, red: q<0.05 significant after BH correction, orange: nominal significant (p<0.05) and black: not significant.

Table S2: Vaccination Details for Phase II Participants

ID	Vaccine Dose 1	Vaccine Dose 2	Vaccine Dose 3	Vaccine Dose 4
2	Pfizer	Pfizer	Moderna	
4	AstraZeneca	AstraZeneca	Pfizer	
5	Janssen	Janssen		
8	AstraZeneca	AstraZeneca	AstraZeneca	
9	Moderna	Moderna		
16	Pfizer			
27	Pfizer	Pfizer	Pfizer	Moderna
38	Pfizer	Pfizer	Pfizer	
49	Janssen	Pfizer		
50	Pfizer	Pfizer	Moderna	
54	Pfizer	Pfizer		
55	AstraZeneca	AstraZeneca		
58	AstraZeneca	AstraZeneca		
65	AstraZeneca	AstraZeneca	AstraZeneca	
66	Sinovac	Sinovac		
70	Sinovac	Sinovac		
72	Pfizer	Pfizer		
73	Pfizer	Pfizer		
74	Pfizer	Pfizer		
77	Pfizer	AstraZeneca		
80	Pfizer	Pfizer		
81	Pfizer	Pfizer	Pfizer	Pfizer
85	Pfizer	Pfizer	Pfizer	Pfizer
93	Sinovac	Sinovac	Moderna	
94	Janssen	Janssen		
95	Moderna	Moderna		
98	Sinovac	Pfizer	Moderna	
109	Moderna	Moderna		
111	AstraZeneca	AstraZeneca		
